# A Novel Phenotype of Junctional Epidermolysis Bullosa with Transient Skin Fragility and Predominant Ocular Involvement Responsive to Human Amniotic Membrane Eyedrops

**DOI:** 10.3390/genes12050716

**Published:** 2021-05-11

**Authors:** Daniele Castiglia, Paola Fortugno, Angelo Giuseppe Condorelli, Sabina Barresi, Naomi De Luca, Simone Pizzi, Iria Neri, Claudio Graziano, Diletta Trojan, Diego Ponzin, Sabrina Rossi, Giovanna Zambruno, Marco Tartaglia

**Affiliations:** 1 Laboratory of Molecular and Cell Biology, IDI-IRCCS, 00167 Rome, Italy; p.fortugno@idi.it (P.F.); n.deluca@idi.it (N.D.L.); 2 Genodermatosis Unit, Genetics and Rare Diseases Research Division, Bambino Gesù Children’s Hospital, IRCCS, 00165 Rome, Italy; agiuseppe.condorelli@opbg.net (A.G.C.); giovanna.zambruno@opbg.net (G.Z.); 3 Genetics and Rare Diseases Research Division, Bambino Gesù Children’s Hospital, IRCCS, 00146 Rome, Italy; sabina.barresi@opbg.net (S.B.); simone.pizzi@opbg.net (S.P.); marco.tartaglia@opbg.net (M.T.); 4 Department of Experimental, Diagnostic and Specialty Medicine, Division of Dermatology, S. Orsola-Malpighi University Hospital, 40138 Bologna, Italy; iria.neri@tin.it; 5 Medical Genetics, S. Orsola-Malpighi University Hospital, 40138 Bologna, Italy; claudio.graziano@unibo.it; 6 Treviso Tissue Bank Foundation, 31100 Treviso, Italy; dtrojan@fbtv-treviso.org; 7 The Veneto Eye Bank Foundation, 30174 Venice, Italy; diego.ponzin@fbov.it; 8 Pathology Unit, Department of Laboratories, Bambino Gesù Children’s Hospital, IRCCS, 00165 Rome, Italy; sabrina2.rossi@opbg.net

**Keywords:** hypomorphic *LAMB3* allele, laminin-332 assembly, corneal erosions, keratinocyte adhesion

## Abstract

Junctional epidermolysis bullosa (JEB) is a clinically and genetically heterogeneous skin fragility disorder frequently caused by mutations in genes encoding the epithelial laminin isoform, laminin-332. JEB patients also present mucosal involvement, including painful corneal lesions. Recurrent corneal abrasions may lead to corneal opacities and visual impairment. Current treatments are merely supportive. We report a novel JEB phenotype distinguished by the complete resolution of skin fragility in infancy and persistent ocular involvement with unremitting and painful corneal abrasions. Biallelic *LAMB3* mutations c.3052-5C>G and c.3492_3493delCG were identified as the molecular basis for this phenotype, with one mutation being a hypomorphic splice variant that allows residual wild-type laminin-332 production. The reduced laminin-332 level was associated with impaired keratinocyte adhesion. Then, we also investigated the therapeutic power of a human amniotic membrane (AM) eyedrop preparation for corneal lesions. AM were isolated from placenta donors, according to a procedure preserving the AM biological characteristics as a tissue, and confirmed to contain laminin-332. We found that AM eyedrop preparation could restore keratinocyte adhesion in an in vitro assay. Of note, AM eyedrop administration to the patient resulted in long-lasting remission of her ocular manifestations. Our findings suggest that AM eyedrops could represent an effective, non-invasive, simple-to-handle treatment for corneal lesions in patients with JEB and possibly other EB forms.

## 1. Introduction

Junctional epidermolysis bullosa (JEB) is a rare genodermatosis typified by skin and mucous membrane fragility and blister formation occurring within the lamina lucida of the epithelial basement membrane zone (BMZ) [1]. JEB is clinically and genetically heterogeneous. Biallelic mutations in seven genes (i.e., *LAMA3*, *LAMB3* and *LAMC2*, encoding laminin-332 (LM332, α3β3γ2); *COL17A1*, encoding type XVII collagen (COLXVII); and *ITGA6*/*ITGB4* and *ITGA3*, encoding α6β4 integrin and α3 integrin subunit, respectively) have been associated with different disease subtypes, which range from severe and early lethal conditions to localized variants with limited skin and mucosal involvement [2].

LM332 is an extracellular matrix glycoprotein of the epithelial basement membrane (BM), which is highly abundant in the skin and mucous membranes of the eye, upper respiratory, gastrointestinal and genito-urinary tracts [1]. Its chain subunits, α3 (200 kDa), β3 (140 kDa) and γ2 (155 kDa), are shaped in a disulphide-bonded coiled-coil cross with one long arm and three short arms. Epithelial cells synthesize and secrete the assembled heterotrimer, which is proteolytically cleaved in the α3 and γ2 chains after secretion [3,4]. Within the BMZ, LM332 bridges the keratinocyte transmembrane proteins α6β4 integrin and COLXVII with the anchoring fibril constituent collagen VII (COLVII), thus ensuring epithelial adhesion to the underlying basal membrane and mesenchyme [1]. The amount of LM332 produced by epithelial cells with pathogenic variants in the *LAMA3*, *LAMB3* or *LAMC2* genes is the major determinant of JEB phenotype severity [5]. The severe JEB subtype results from absent LM332, while intermediate or localized JEB forms associate with variably reduced or even normal amounts of mutant LM332 [6,7,8,9].

Ocular involvement is common in JEB, with corneal disease being reported in 35–40% of the patients [10,11,12]. Of note, dominant mutations in COLXVII cause a peculiar form of corneal dystrophy with no skin fragility known as epithelial recurrent erosion dystrophy (ERED) [13,14]. Recurrent corneal abrasions can lead to corneal opacities and eventually to visual loss in both JEB and ERED. Current treatments are merely supportive and include regular use of artificial tears and lubricating ointments, aimed at reducing friction and thus limiting disease recurrence. An alternative therapeutic approach described in severe EB cases is based on the transplantation of human amniotic membrane (AM) [15]. AM is a fetal tissue comprised of an epithelial layer, a BM and stroma. It contains antiangiogenic and anti-inflammatory molecules able to limit ocular damage after injuries, promote ocular surface repair, and endowed with tectonic capacities [16,17]. For these reasons, AM transplantation has been used as a temporary patch or permanent graft in several ocular surface diseases and reconstruction procedures for many years [16,17]. 

We report a novel phenotype of JEB characterized by transient neonatal skin fragility and unremitting corneal involvement due to compound heterozygous *LAMB3* mutations, one being a hypomorphic variant. Treatment with an AM eyedrop preparation containing LM332 led to long-lasting remission of corneal lesions paralleled by in vitro reversion of keratinocyte adhesion defect.

## 2. Materials and Methods

### 2.1. Immunofluorescence and Ultrastructural Analyses and Cell Cultures

Patient skin biopsies were obtained and processed for (i) immunofluorescence antigen mapping (IFM) and ultrastructural examination, and (ii) keratinocyte (KC) cultures, as described in References [18,19]. Immunofluorescence laminin 332 expression levels were obtained by measuring optical counts of staining intensity, using ImageJ software. Specifically, the Corrected Total Cell Fluorescence (CTCF) of several selected regions at the dermal–epidermal junction was calculated by using the following formula: CTCF = Integrated Density–(Area of selected region × Mean fluorescence of background readings). Then, the average of CTCF values from different patient’s images was compared to that of control samples (3–10 selected regions for each IF image, 4 different IF images for each sample). *p*-value was calculated by using the *t*-test (2 tails; type 2). For ultrastructural examination, ultrathin sections of the patient’s biopsy and one control biopsy of clinically normal skin from an age-matched subject were viewed with a Jeol JEM-1400 transmission electron microscope (JEOL Ltd., Tokyo, Japan). For hemidesmosome counts, electron micrographs of overlapping fields of epidermal BMZ from the patient and control were printed and the length of the BMZ, excluding tracts associated with melanocytes, was measured by using ImageJ software. The number of hemidesmosomes, defined as discrete electron dense structures along the keratinocyte plasma membrane, was determined by manual counting. At least 100 µm of BMZ was evaluated in each sample.

### 2.2. AM Eyedrop Preparation

Selection of donor placentas and separation and processing of AM to obtain eyedrops are detailed in Appendix A. The AM eyedrop preparation was aliquoted and stored at –80 °C until delivery to the patient.

### 2.3. DNA and RNA Studies

Genetic analysis was carried out on genomic DNA from blood by clinical exome sequencing (Trusight One panel, Illumina, San Diego, CA, USA), using a NextSeq 500 platform, with 20× average sequencing coverage. Read files were obtained from the Illumina platform through the manufacturer’s proprietary software (Illumina VariantStudio Software v3.0, Illumina, San Diego, CA, USA). Variant validation and segregation analysis were performed by Sanger sequencing. In silico analysis of the functional impact of the identified LAMB3 mutations was performed by NNSPLICE (0.9 version) (https://www.fruitfly.org/seq_tools/splice.html, accessed on 24 January 2019) and Combined Annotation Dependent Depletion (CADD 1.3) (http://pec630.rockefeller.edu:8080/MSC/, accessed on 23 April 2021) prediction software program. Total RNA from patient keratinocytes was reverse transcribed (Superscript III RT, Thermo Fisher Scientific, Carlsbad, CA, USA) and amplified with oligonucleotides 5’-GCTGAGGCTGAGGAAGCCAG-3’ (forward primer) and 5’-TGTAACTGTCCCATTGGCTC-3’ (reverse primer) to generate a fragment encompassing the two parental variants [20]. The resulting PCR product was then cloned in a pCR2.1 vector (TA cloning kit, Thermo Fisher Scientific), and individual subclones were sequenced.

### 2.4. Immunoprecipitation and Immunoblotting Assays

Immunoprecipitation (IP) of LM332 was performed on conditioned media from patient and healthy control KCs (PKs and NHKs, respectively) using monoclonal antibody (mAb) GB3, which binds to an epitope in the LM332 heterotrimer, or mAb K140, which recognizes a motif within the N-terminus of the β3 chain short arm [21]. Conditioned media were collected from sub-confluent cells grown for 18–24 h in KC medium containing 2% of fetal bovine serum (FBS) and concentrated using Amicon Ultra-15 Centrifugal Filter Units (10.000 MW cutoff) (Merck Millipore, Burlington, MA, USA). Then, equal amounts of immunoprecipitated molecules were probed by immunoblotting. Samples were run under reducing conditions, using 4–6% polyacrylamide gels, and immunoblotted with a polyclonal antibody that recognizes epitopes in the middle portion of the β3 chain long arm (Cod. TA308917, Origene, Rockville, MD, USA). Detection was performed by using Amersham ECL Prime (GE Healthcare Life Sciences, Little Chalfont, UK). LM332 content in AM eyedrops was confirmed by dot blot analysis (Supplementary Materials Figure S2a). Specific signals were revealed by chemiluminescence, using Bio-Rad ChemiDoc Imaging System, and quantified by using ImageLab software (Bio-Rad, Hercules, CA, USA). 

### 2.5. Trypsin-Detachment Assays

To evaluate the effects of AM eyedrops on the adhesive properties of PKs and NHKs, 96-well plates were coated overnight (ON), at 37 °C, with 30 µL of AM eyedrops. Controls were represented by 96-well plates coated with PBS or with human recombinant LM332 (r-LM332) (Biolamina, Sunbyberg, Sweden), at the concentration of 0.5 µg/cm^2^, following the manufacturer’s coating protocol. The day after, AM eyedrops, r-LM332 and PBS were removed from the wells, and PKs and NHKs were seeded at a density of 4 × 10^4^ cells/well in Keratinocyte Growth Medium (KGM) (Lonza, Basel, Switzerland). Twenty-four hours after seeding, KCs were incubated with trypsin solution (0.05% trypsin, EDTA 0.02%) for 0–7 min. Trypsin detachment assay was performed as described in Reference [22]. 

## 3. Results

### 3.1. A Novel JEB Phenotype with Transient Skin Fragility and Severe Ocular Involvement

**Figure 1:**
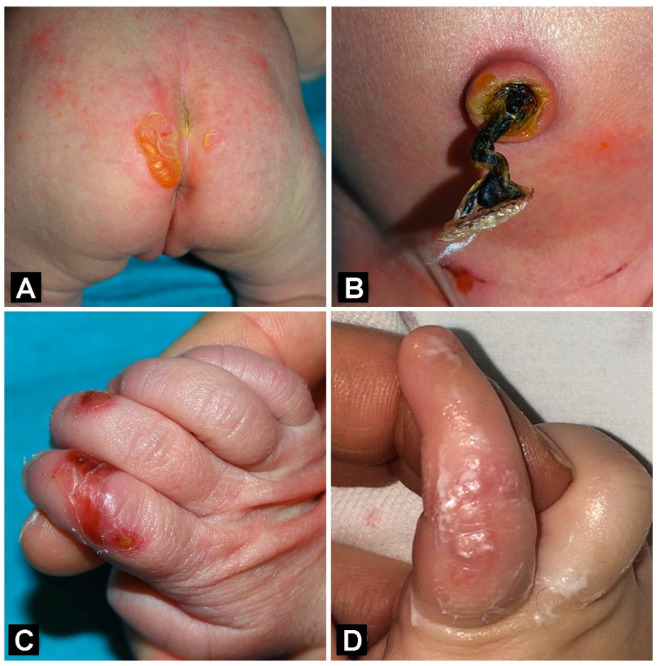
Junctional epidermolysis bullosa patient’s clinical features in infancy. Clear blisters and erosions of the buttocks, umbilicus and pubic region in the first days of life (**A**,**B**). Finger erosions and crusts, and residual atrophy with milia at age of 1 month and 4 months, respectively (**C**,**D**).

A female full-term neonate, the first child of healthy non-consanguineous parents, presented at birth with clear blisters at trauma-exposed sites (diaper area, umbilicus, elbows, knees and extremities) (Figure 1A,B), subungual hemorrhages and nail dystrophies. Lesions healed with minimal atrophic scarring and milia formation (Figure 1C,D).

**Figure 2:**
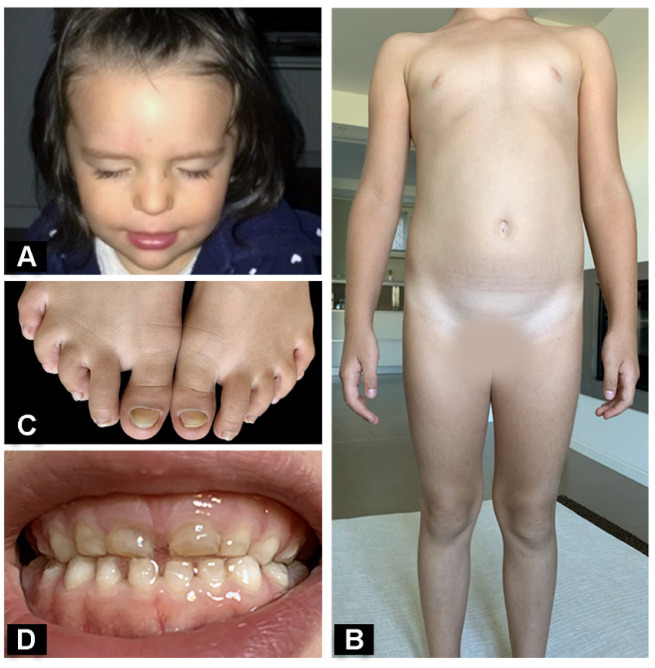
Junctional epidermolysis bullosa patient’s evolution over time. A typical image of the patient keeping her eyes closed because of pain at eyelid blinking at age 2 years (**A**). Physical appearance of the patient at age 6 years: absence of active skin lesions (**B**), with modest toenail dystrophy (**C**) and enamel pitting (**D**).

Skin fragility markedly improved already in infancy, while recurrent painful corneal abrasions manifested by 15 months of age. In addition, roundish papular lesions with a central whitish deposit presented on fingers. Starting from the second year of life, skin fragility completely resolved, while the number and severity of corneal erosions gradually increased reaching an almost weekly frequency, despite regular use of eye lubricants. The parents reported that corneal lesions were more frequent in winter and linked worsening to dry and cold weather. The patient was addressed to us at the age of 3 years with a suspected diagnosis of inherited epidermolysis bullosa to perform the complete laboratory workup for disease confirmation and subtyping. Physical examination showed localized atrophic scars of knees and elbows, three papular lesions on fingers (Supplementary Materials Figure S1A), minimal toenail dystrophies and enamel pitting. The child also presented photophobia and complained of severe eye pain, keeping her eyes closed most of the time (Figure 2A).

**Figure 3:**
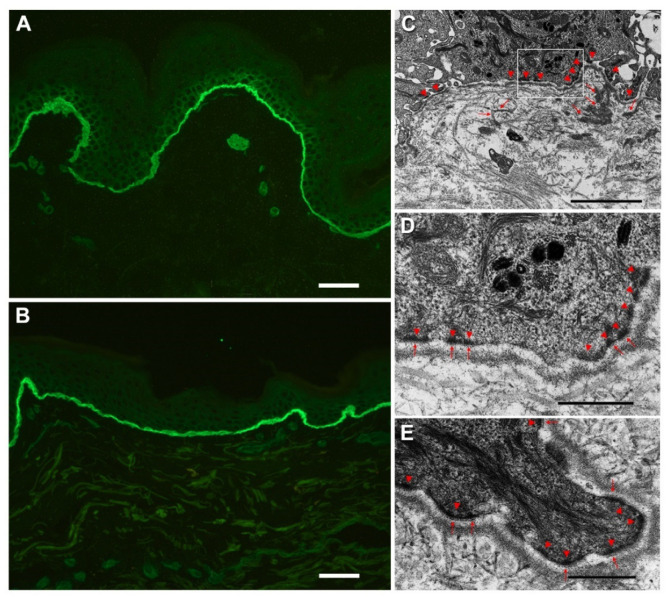
Patient’s immunofluorescence, ultrastructural and molecular genetic characterization. Immunofluorescence antigen mapping with monoclonal antibody GB3 shows absence of skin blistering and expression of laminin-332 in patient’s skin (**A**) comparable to normal control skin (**B**). Note the faint cytoplasmic labeling in basal keratinocytes of patient’s skin. Ultrastructural appearance of patient’s skin: a few hypoplastic hemidesmosomes (arrowheads in (**C**) and (**D**), together with short reduplications of the basal lamina (arrows in (**C**)), are visible. A higher magnification of the inset in (**C**) is shown in (**D**): patient’s hemidesmosomes appear of small size with attenuated or absent sub-basal dense plates (arrows in **D**), as compared with hemidesmosomes in normal skin of a control individual ((**E**) arrowheads indicate hemidesmosomes and arrows denote sub-basal dense plates). Bar = 25 µm in (**A**,**B**); 2 µm in (**C**); 500 nm in (**D**,**E**).

The hematoxylin–eosin staining (Supplementary Materials Figure S2) and IFM of a perilesional frozen skin biopsy showed absence of microblistering. Expression of major protein components of the epidermal adhesion complex (keratins 5 and 14; plectin; α6β4 integrin; LM332; and collagens IV, VII and XVII) was comparable to healthy control skin (Supplementary Materials Figure S3). A faint basal keratinocyte staining was noted only with anti-LM332 mAb (Figure 3A,B). Computational quantification of the LM332 staining intensity at the dermal–epidermal junction did not show significant differences in protein expression between patient and a normal control (Supplementary Materials Figure S4).

Skin ultrastructural examination revealed microblistering within the lamina lucida of the BMZ, together with a reduced number of hemidesmosomes (n = 11.9 per 10 µm of patient’s epidermal BMZ length versus 21.7/10 µm in age-matched control skin) (Figure 3 C,D). In addition, the vast majority (>80%) of the patient’s hemidesmosomes appeared of small size with attenuated to absent sub-basal dense plates (Figure 3D) as compared with normal skin hemidesmosomes (Figure 3E). Short duplications of the lamina densa were frequently observed in the papillary dermis (Figure 3C). Surgical excision of a finger papule revealed calcinosis in the superficial and mid-dermis with an overlying acanthotic and hyperkeratotic epidermis (Supplementary Materials Figure S1B,C).

### 3.2. Compound Heterozygosity for Readthrough and Splice Site LAMB3 Variants Underlies a Novel JEB Phenotype

**Figure 4:**
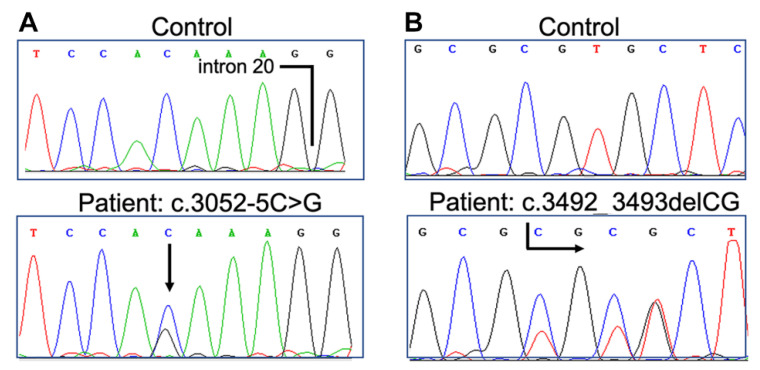
Sequence electropherograms showing LAMB3 mutations c.3052-5C>G (**A**) and c.3492_3493delCG (**B**). Wild-type sequences from a normal control are shown for comparison.

Interrogation of the exome datasets revealed two heterozygous putative pathogenic variants in *LAMB3*, c.3052-5C>G and c.3492_3493delCG, which had not previously been reported in public databases (Figure 4A,B). The c.3052-5C>G substitution was inherited from the father and affected the intron 20 acceptor site. This transversion was predicted to create a new acceptor site five nucleotides upstream the natural one (tctttccacaaag → tctttccagaaag, score 0.98), thus resulting in the insertion of a Lys residue, followed by a frameshift leading to protein truncation (p.Val1018Glyfs*21). The c.3492_3493delCG frameshift in exon 23 was inherited from the mother and was predicted to lead to stop codon readthrough (p.Val1165Alafs*56), resulting in a 47 residue–longer mutant β3 chain. CADD score for mutations c.3492_3493delCG and c.3052-5C>G was 35 (high impact for the gene) and 5.023 (low impact for the gene), respectively, with 6.464 being the mutation significant cutoff for *LAMB3* (95% CIs).

### 3.3. c.3052-5C>G Results in Residual Secretion of Wild-Type LM332

**Figure 5:**
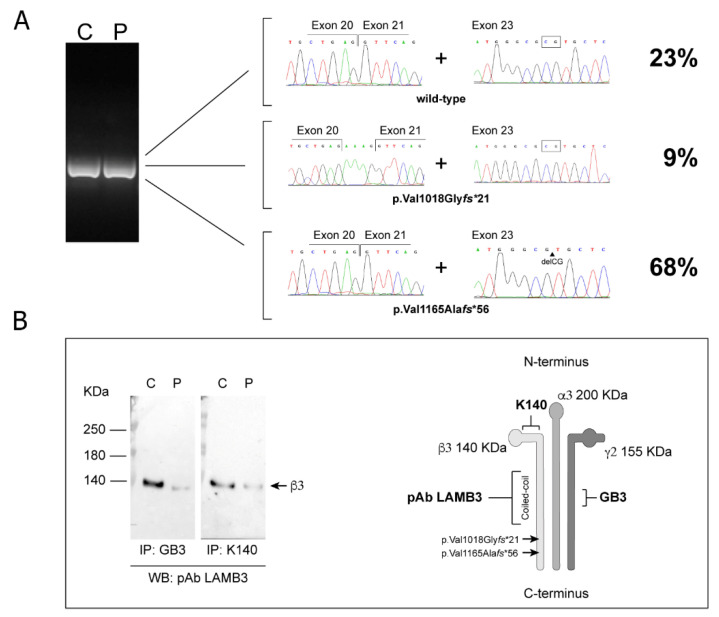
Gene expression analysis. Visualization of the cDNA band amplified from patient’s RNA (P) using primers encompassing both mutation sites. No altered size can be appreciated in comparison to the control band (C) ((**A**) left panel). Sequencing across the mutation sites of subcloned cDNA molecules (n = 22) identifies transcripts (68%) linked to the c.3492_3493delCG mutation only (lower right panel), transcripts (9%) that held the intron 20 insertion (aaag) but not the c.3492_3493delCG deletion (middle right panel), and transcripts (23%) that are wild-type at both mutation sites (upper right panel). Immunoprecipitation (IP) of laminin-332 (LM332) from keratinocyte (KCs) conditioned media from patient (P) and a normal control (C), using the monoclonal antibodies (mAb) GB3 and K140. Equal amounts of immunoprecipitated LM332 were loaded onto 4–6% polyacrylamide gels under reducing conditions. Immunoblotting analysis was performed by using the polyclonal antibody (pAb) Cod. TA308917 (Origene), which recognizes epitopes in the middle portion of the β3 chain long arm ((**B**) left panel). Scheme of LM332 structure that shows the position of the epitopes recognized by the antibodies and mutation sites ((**B**) right panel). For simplicity the LM332 structure lacks the G domain in the α3 C-terminus.

The functional consequences of the splice site change were evaluated at both mRNA and protein levels. Twenty-two subclones obtained by cloning of an apparently unique cDNA product amplified with primers encompassing the two identified *LAMB3* variants were sequenced. The analysis showed that 15 subclones (68%) contained only the c.3492_3493delCG deletion, two (9%) were characterized by the retention of four intronic nucleotides and five (23%) corresponded to the wild-type coding sequence (Figure 5A). We reasoned that wild-type sequence not including the maternal c.3492_3493delCG deletion originated from a “leaky” splicing regulation due to the paternal c.3052-5G>C substitution, supporting the occurrence of residual wild-type LM332 in patient skin and cultured keratinocytes. To confirm this prediction, protein immunoblotting assays was performed to detect β3 chains in immunoprecipitated LM332 from patient cell conditioned medium. No product corresponding to the elongated or to the truncated LM332 was identified, indicating accelerated degradation of these mutant forms. However, a band corresponding to the wild-type β3 polypeptide (140 kDa) was observed, although substantially reduced compared to control keratinocytes (10%) (Figure 5B). Taken together, these analyses demonstrated that patient keratinocytes secrete reduced amounts of wild-type LM332 due to the hypomorphic c.3052-5C>G *LAMB3* allele.

### 3.4. An AM Eyedrop Preparation Induces Long-Lasting Remission of Corneal Abrasions and Increases Patient Keratinocyte Adhesion.

Having established that LM332 in our patient was quantitatively affected and its residual amount (10%) was able to rescue skin blistering but not sufficient to prevent recurrent corneal lesions, the affected child was referred to the Veneto Eye Bank Foundation. Ophthalmological evaluation revealed a moderate ocular surface inflammation in both eyes with conjunctival hyperemia, mild epithelial edema and an epithelial lesion of 2 square millimeters in the right eye, with symptoms of photophobia and severe discomfort. No relevant corneal scarring could be observed. The patient was treated with the AM eyedrop preparation five times a day, for a course of two months, resulting in a complete, long-lasting remission of corneal lesions and ocular symptoms. Notably, corneal abrasions recurred 15 months later, but the repeated application of the same preparation for one month confirmed able to induce remission of corneal manifestations for the subsequent 12 months, till present. The child, who is now 6.5 years old, does not show any skin fragility and only presents enamel pitting and mild toenail dystrophy (Figure 2B–D). 

**Figure 6:**
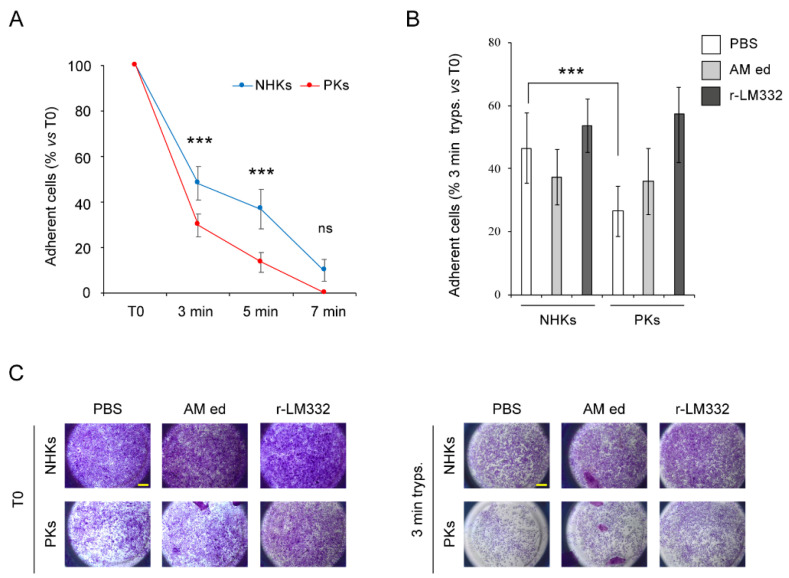
Amniotic membrane (AM) eyedrops rescue patient primary keratinocyte adhesion defect. Figure shows the results of the trypsin-based detachment assay. Briefly, after 24 h from seeding, confluent primary keratinocytes from patient (PKs) and normal controls (NHKs) (n = 2) were treated with trypsin/EDTA solution (0.05%/0.02%). After trypsinization, residual adherent cells were stained with a 0.5% crystal violet (CV) solution and lysed with 1% sodium dodecyl sulfate (SDS) to release dye. The absorbance of the dye solution was measured by spectrophometer at 595 nm. PKs and NHKs were grown on non-coated 96-well plates (i.e., basal condition) and subjected to trypsinization for 3, 5 and 7 min. T0 indicates cells untreated with trypsin, stained with CV and lysed by SDS 1%. Histogram indicates the percentage of trypsin resistant, adherent cells ± standard deviation (SD) with respect to T0. Data were obtained by using at least three replicates for each experimental condition (**A**). PKs and NHKs (n = 2) were seeded on culture wells coated with AM eyedrops (AM ed) or recombinant laminin-332 (r-LM332). Culture wells coated with PBS were used as control. The day after, KCs were subjected to 3 min of trypsination. Histogram indicates the percentage of trypsin resistant, adherent cells ± SD with respect to T0. Data were obtained by using at least three replicates for each experimental condition (**B**). Representative pictures showing PKs and NHKs at T0 or treated with trypsin for 3 min, in the presence or absence of AM ed and r-LM332 coating, and then stained with CV solution. Scale bar = 500 µm (**C**). *** *p* ≤ 0.005; ns = non-significant (*p* > 0.05).

In view of the clinical efficacy of the AM eyedrop preparation in preventing corneal lesion recurrence in our patient, we wanted to investigate their effect in vitro on PKs functional properties. As AM shares most protein adhesion components with the skin [23], we confirmed the presence of LM332 within the AM eyedrop preparation by dot-blot experiments (Supplementary Materials Figure S5A). Then, the adhesion properties of PKs and NHKs were evaluated in basal conditions (T0) and using a trypsin-based detachment assay. As expected, PKs showed reduced adhesion at T0 (Supplementary Materials Figure S5B), and a significant increase in detachment of PKs (% of residual adherent cells: 29.6 ± 5) compared to NHKs (48 ± 7.4) was detected already after three min of trypsinization (*p* < 0.005). Similarly, a five-min trypsin treatment resulted in a significantly increased detachment of PKs (13 ± 4.2% of adherent cells) as compared with NHKs (37 ± 8.8% of adherent cells) (*p* < 0.005) (Figure 6A). These results confirmed that the reduced amount of LM332 produced by PKs negatively impacts their adhesive properties in vitro. Cells were then seeded onto 96-well plates coated with the AM eyedrop preparation or r-LM332 and subjected to the trypsin-based detachment assay. PBS-coated wells were used as control. Strikingly, after 3 min of trypsinization the percentage of adherent PKs and NHKs resulted comparable (36 ± 9% and 37 ± 10% residual adherent cells, respectively) (*p* = 0.41) in AM preparation-coated wells (Figure 6B,C). As expected, r-LM332 coating was able to restore and enhance the adhesive properties of PKs and NHKs (% of residual adherent cells: 57 ± 11 vs. 54 ± 8, respectively; *p* = 0.28) (Figure 6B,C). The effects of AM preparation on cell adhesion could not be detected anymore after 5 min of treatment, likely due to the trypsin-mediated degradation of AM proteins, including the exogenous LM332 [24].

## 4. Discussion

We presented a unique phenotype of JEB due to a previously unreported hypomorphic *LAMB3* variant found in association with a loss-of-function *LAMB3* mutation, resulting in residual secretion of LM332. This distinctive presentation was characterized by the resolution of skin fragility by the second year of life and a persistent ocular phenotype with unremitting corneal lesions. An additional feature was the presence of dystrophic calcinosis cutis papular lesions, which have been described only in dystrophic EB to date [25]. Genotype–phenotype correlations drawn from published cases with localized or intermediate JEB phenotypes predict that LM332 chain mutations allowing the synthesis and secretion of reduced or even normal amounts of the mutant heterotrimeric molecule prevent early lethality and are compatible with normal life expectancy [5]. In the large majority of cases, such mutations affect splice sites or codon sequence and cause in-frame deletions or single amino acid changes in LM332 subdomains [6,7,8,9,20]. In frame deletions within the coiled coil domain always spare the LM332 C-terminal ends that comprise sequences crucial for heterotrimer assembly and stabilization [26]. Indeed, truncation or alterations of these C-terminal sequence elements usually result in null protein products [27]. Both the p.Val1018Glyfs*21 and p.Val1165Alafs*56 mutant chains here described belong to the latter category. Our findings indicate that these mutant polypeptides do not form heterotrimers, as no β3 chains with altered size could be detected in LM332 molecules immunoprecipitated with a mAb (GB3) that recognizes assembled heterotrimers. Moreover, the p.Val1018Glyfs*21 is also most likely targeted at mRNA level by nonsense mediated mRNA decay [28]. Importantly, the p.Val1165Alafs*56 mutant chain (47 aa longer) generated by the readthrough mutation c.3492_3493delCG is similar in length to the elongated mutant β3 chain (56 aa longer) that resulted from another, previously reported, readthrough mutation causing a severe JEB phenotype [29]. Consequently, the JEB phenotype of our patient should be ascribed to the residual synthesis of wild-type LM332 by the hypomorphic c.3052-5C>G allele. Of note, the low CADD score for this non-canonical splicing variant is consistent with its leaky effect. Nevertheless, the amount of wild-type LM332 has relevant phenotypic implications [30]. While LM332 secreted by cultured keratinocytes of our patient was reduced by 90%, the levels of LM332 detected in patient skin in childhood appeared almost comparable to those of normal control skin. We hypothesize that wild-type LM332 released by basal cells in vivo is stable and accumulates at the BMZ, thus contributing to restore skin resilience soon after birth. In addition, splicing modulation favoring the wild-type/full length transcript might occur in postnatal life and contribute to increase wild-type/full size LM332 amount in the skin and thus to rescue skin fragility, as already described for different leaky splicing mutations in *LAMB3* and ITGB4 [31,32]. Finally, the frequency of the wild-type spliced transcripts observed in primary keratinocytes in vitro may not exactly reflect the in vivo situation, and splicing efficiency may vary in different tissue contexts [33]. 

While skin fragility resolved by the second year of life in our patient, recurrent corneal erosions continued to develop during childhood. Corneal lesions represent a well-known manifestation of JEB: they have been reported to occur in about 50% of severe JEB patients and about 25% of other JEB subtypes, with onset already in infancy and progressive course [34]. In different JEB subtypes, defects in all LM332 chains, COLXVII as well as α6β4 integrin can associate to corneal involvement, in addition to skin fragility [5,35,36]. Major structural protein components (i.e., BP230, plectin, COLXVII, α6β4 integrin, LM332 and collagen VII) of the anchoring complexes linking intracellular keratin intermediate filament cytoskeleton of basal epithelial cells through hemidesmosomes and anchoring fibrils to the BMZ and underlying stroma are shared between the skin and cornea [23]. These include proteins mutated in JEB, as well as in dystrophic EB, and explain the occurrence of fragility in both epithelia. Despite these similarities, differences in structure and protein components exist, allowing these tissues to accomplish their specific functions [23]. To our knowledge, remission of skin fragility accompanied by persistent and progressive ocular involvement has not been described in JEB. Specific to the phenotype of our patient, it might be hypothesized that unremitting corneal wounding, together with a more rapid turn-over of the corneal BM, underlies the ocular phenotype of the child. In addition, tissue specific differences in splicing modulation in the cornea versus the skin might result in lower amounts of wild-type β3 transcripts and, thus, decreased LM332 levels [33]. 

To date, there are no effective therapies for EB ocular disease. Current treatments are merely supportive and include artificial tears and lubricating ointments, which do not prevent disease recurrence and frequently also do not allow for pain control, as in the present case. Due to inefficacy of conventional care and risk of visual loss, treatment with AM eyedrops was started with impressive clinical outcome: a single cycle resulted in complete and long-term remission of corneal lesions. Furthermore, AM eyedrop efficacy was maintained over time as renewed application following relapse led to long-lasting phenotypic corneal rescue associated with pain disappearance and highly improved quality of life. In addition to sharing most adhesive protein components with skin, the AM is endowed with (i) anti-inflammatory effects owing to the production of anti-inflammatory factors, such as hyaluronic acid and suppression of proinflammatory cytokines; (ii) anti-fibrotic and anti-scarring properties, in particular due to the downregulation of TGF-β signaling; and (iii) low immunogenicity. Due to its properties, AM has been used for treatment of a variety of pathological conditions affecting the skin and eye, from leg ulcers to burns and mucous membrane pemphigoid, without the need for concomitant immunosuppressive treatment [15,17,37,38,39,40,41]. AM grafting has also been reported for the treatment of chronic skin ulcers and severe eye lesions, in particular symblepharon and corneal opacities, in single patients affected with dystrophic and junctional EB [42,43,44,45,46,47]. Recent studies have shown that the topical application of AM homogenate/extract may have a comparable effect in promoting epithelialization, decreasing inflammation and preventing corneal neovascularization [48,49]. These studies have extended the indication of AM, allowing the continuous application of the tissue in an outpatient setting or at the patient’s home, with the advantage of adding the lubricating and hydrating effect of a biological tear substitute, as with the autologous serum eyedrops. Of note, our in vitro findings show that AM eyedrops rescue defective adhesion of patient keratinocytes to levels comparable to normal control cells. Restoration of adhesive function represents a crucial step for successful JEB treatment. The anti-inflammatory and anti-fibrotic properties of AM could have also contributed to the impressive and long-lasting ocular disease remission. Overall, our findings suggest that AM eyedrops could represent an effective, non-invasive, simple-to-handle treatment for corneal lesions in patients with JEB and possibly other EB forms due to extracellular matrix component deficiency.

## 5. Conclusions

In conclusion, we depicted a novel JEB phenotype characterized by remission of skin fragility accompanied by persistent and progressive ocular involvement. We linked the transient skin fragility to a hypomorphic *LAMB3* allele. Thus, our findings contribute to expand the phenotypic and genotypic spectrum of JEB and have relevant translational implications since they prospect the possible use of AM eyedrops as effective, non-invasive, simple-to-handle treatment for eye manifestations in JEB individuals.

## Data Availability

The data presented in this study are available on request from the corresponding author. The data are not publicly available, due to privacy reasons.

## Supplementary Materials

The following are available online at https://www.mdpi.com/article/10.3390/genes12050716/s1. Figure S1: Dystrophic calcinosis cutis papular lesions. Figure S2: Hematoxylin–eosin staining of a frozen skin biopsy. Figure S3: Immunofluorescence antigen mapping in patient and control skin. Figure S4: Immunofluorescence LM332 expression level quantification of staining intensity. Figure S5: Laminin-332 content in AM eyedrops and keratinocyte adhesion assay.

## Appendix A

Amniotic membrane (AM) eyedrop preparation: The placentas were sourced from donors undergoing elective caesarean delivery after a minimum of 35 weeks of gestation, in compliance with established European regulation (Directive 2004/23/EC of the European Parliament and of the council of 31 March 2004; Commission Directive 2006/17/EC of 8 February 2006, available at https://eur-lex.europa.eu/LexUriServ/LexUriServ.do?uri=OJ:L:2004:102:0048:0058:en:PDF; https://eur-lex.europa.eu/LexUriServ/LexUriServ.do?uri=OJ:L:2006:038:0040:0052:EN:PDF, accessed on 23 April 2021). Contraindications include malignant pathologies, fetal malformation or pathology, plus any positivity for serologic or molecular screening for transmissible diseases. The placentas were processed shortly after retrieval. The AM was carefully detached from the underlying chorion and rinsed with 0.9% NaCl to remove residual blood and debris. The AM was subsequently immersed in antibiotic cocktail solution made of vancomycin 100 μg/mL (Hospira, Naples, Italy), meropenem 200 μg/mL (Fresenius Kabi, Verona, Italy) and gentamicin 200 μg/mL (Fisiopharma, Salerno, Italy), and then it was dissolved in BASE medium (Alchimia, Pordenone, Italy) overnight, at +4 °C. Hence, the AM was cut into fragments and ground in sterile balanced salt solution (1 g wet weight/10 mL BSS). This procedure does not represent a substantial manipulation (Annex I of Regulation EC No 1394/2007, available at https://eur-lex.europa.eu/LexUriServ/LexUriServ.do?uri=OJ:L:2007:324:0121:0137:en:PDF, accessed on 23 April 2021), thereby preserving the AM biological characteristics and the legal status of a tissue processed and distributed according to the EU/national regulation for tissue banking and transplantation (European Directorate for the Quality of Medicines & HealthCare of the Council of Europe (EDQM). Guide to the quality and safety of tissues and cells for human application, 4th Edition 2019, available at https://www.edqm.eu/en/organs-tissues-and-cells-technical-guides, accessed on 23 April 2021).
