# Supplementary material for: A Novel Phenotype of Junctional Epidermolysis Bullosa with Transient Skin Fragility and Predominant Ocular Involvement Responsive to Human Amniotic Membrane Eyedrops
Source: Genes (Basel). 2021 May 11;12(5):716. doi: 10.3390/genes12050716 (PMC8151857; doi:10.3390/genes12050716)
Supplement: Supplementary file 1 [file genes-12-00716-s001.zip › genes-1178210-supplementary.pdf]

**A novel phenotype of junctional epidermolysis bullosa with transient skin fragility and predominant ocular involvement responsive to human amniotic membrane eyedrops**

Daniele Castiglia, Paola Fortugno, Angelo Giuseppe Condorelli, Sabina Barresi, Naomi De Luca, Simone Pizzi, Iria Neri, Claudio Graziano, Diletta Trojan, Diego Ponzin, Sabrina Rossi, Giovanna Zambruno, and Marco Tartaglia

**SUPPLEMENTARY FIGURES**

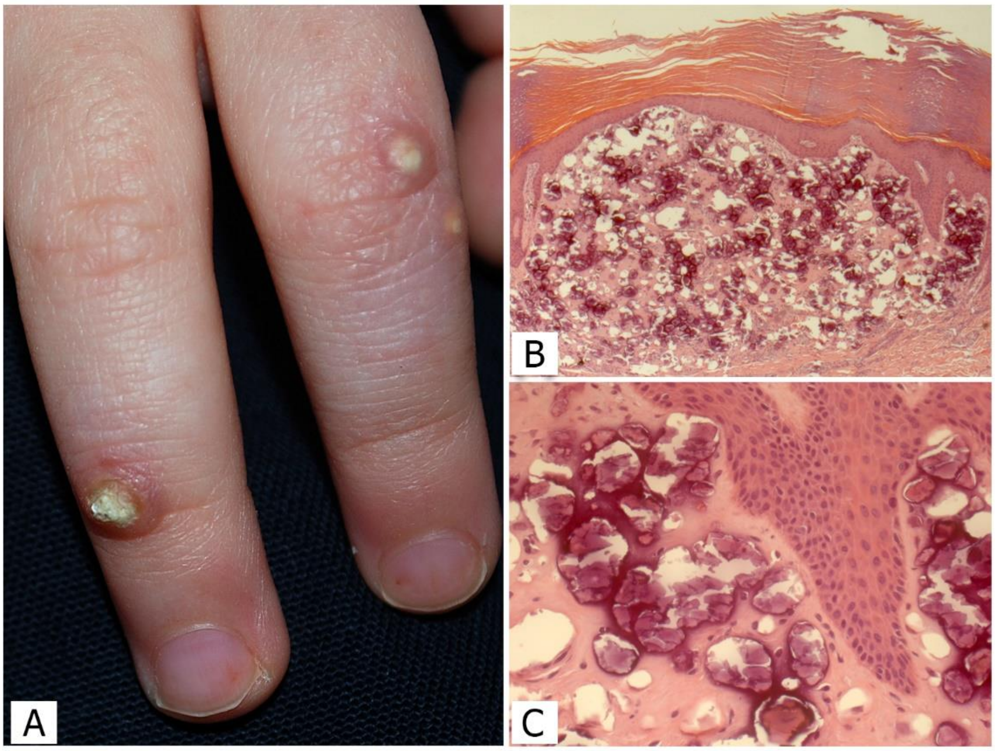


**Supplementary Figure S1**. Three papular lesions with a central whitish material on the second and third right fingers of the patient at age 2.5 years (**A**). Histopathology of a lesion showing dermal calcium deposits appearing as well-circumscribed, intensely basophilic acellular material in the papillary and mid-dermis (**B**) (hematoxylin-eosin staining, original magnification 25X), the epidermis is acanthotic and markedly hyperkeratotic; at higher magnification, the basophilic deposits are surrounded by a darker rim and an eosinophilic hyalinized matrix (**C**) (original magnification 200X).

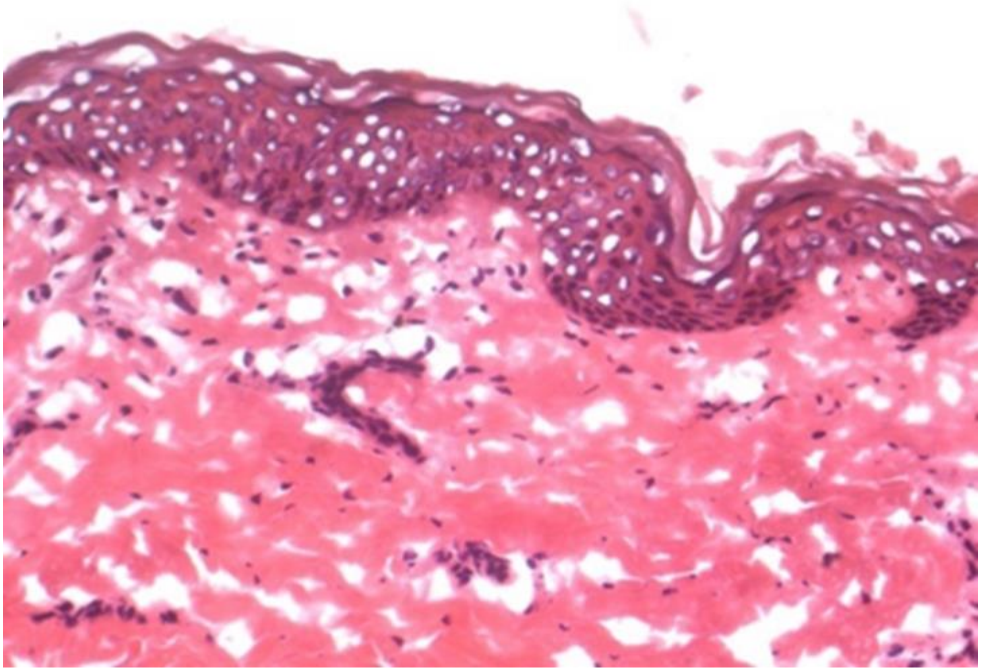

**Supplementary Figure S2**. Hematoxylin-eosin staining of a frozen skin biopsy shows absence of microblistering.

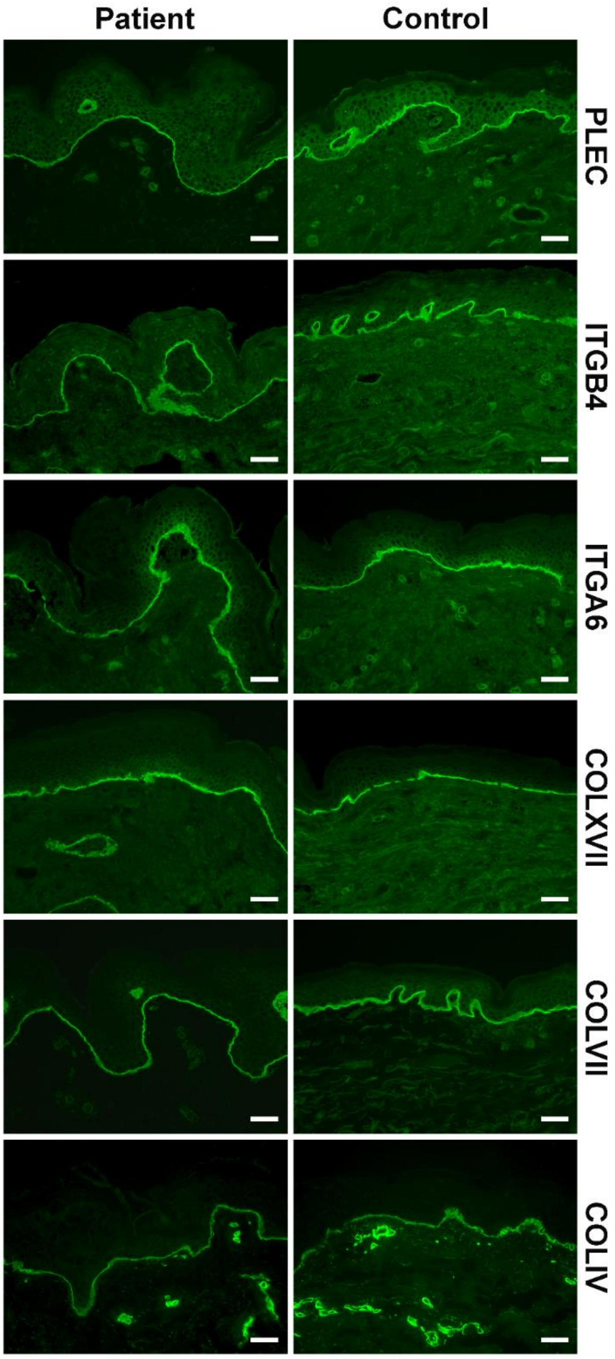


**Supplementary Figure S3.** Expression of the major epidermal adhesion protein components, as determined by immunofluorescence antigen mapping on cryosections of patient and control skin. PLEC, plectin; ITGB4, integrin β4; ITGA6, integrin α6; COLXVII, collagen XVII; COLVII, collagen VII; COLIV, collagen IV. Primary antibodies used are described in reference 18. Bar = 20 µm.

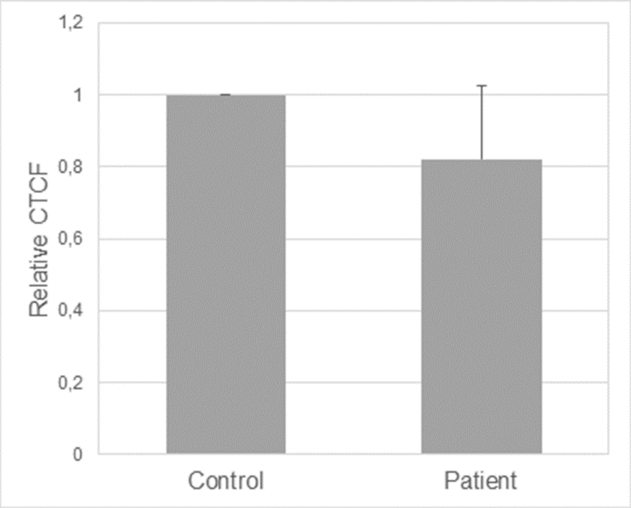


**Supplementary Figure S4.** Laminin-332 expression level in patient and control skin, as determined by ImageJ software quantification of staining intensity. Histograms show the relative Corrected Total Cell Fluorescence (CTCF) measured on images obtained from different laminin-332 stained (GB3 antibody) skin sections (n=4) from the patient and normal control. The CTCF is slightly reduced in the patient compared to the normal control, however this difference is not statistically significant ($P$ = 0.20).

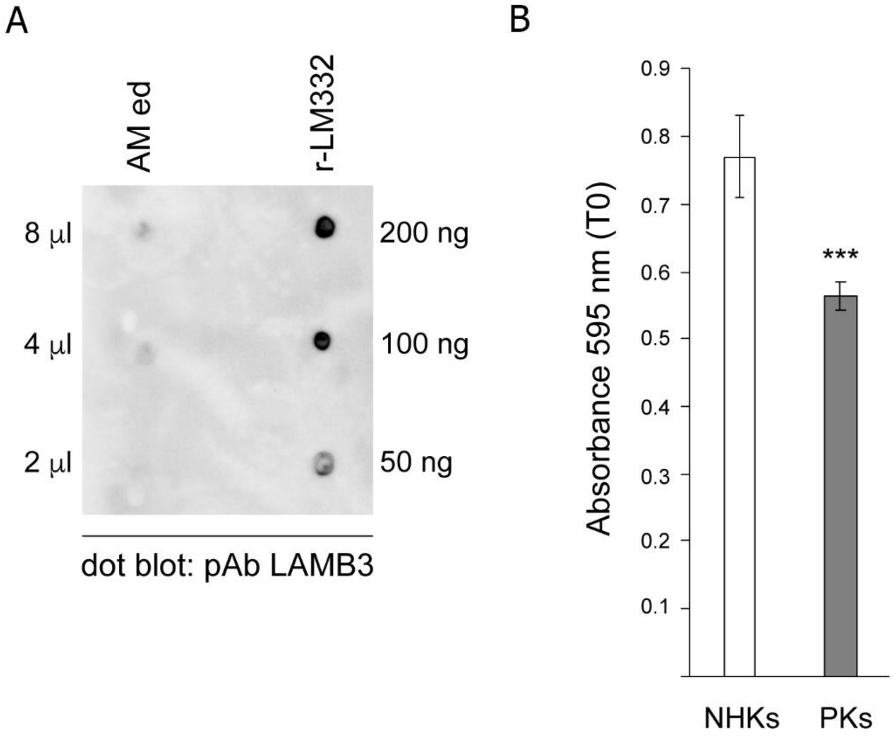


**Supplementary Figure S5**. Dot blot showing laminin-332 (LM332) content in amniotic membrane (AM) eyedrops. Decreasing amounts of AM preparation (AM ed; 8, 4 and 2 µl) and recombinant LM332 (r-LM332; 200, 100 and 50 ng) (Biolamina, Sundbyberg, Sweden) were spotted onto nitrocellulose membrane, let dry out and probed with the anti-laminin β3 polyclonal antibody (pAb)(Cod. TA308917, Origene) (**A**). Primary keratinocytes from patient (PKs) and normal controls (NHKs) were seeded at high density (40,000 cells per well) onto 96-well plates. The day after, cells were stained with crystal violet (CV) and lysed by SDS 1% to release dye. The absorbance of the dye solution was measured by spectrophotometer at 595 nm. Greater absorbance values indicate a higher ability of cells to adhere in basal conditions (no treatment with trypsin) (**B**). Histograms in panel B indicate the absorbance of NHKs and PKs at T0 ± standard deviation. *** $P \leq 0.005$.
